# Differences in Working Memory With Emotional Distraction Between Proficient and Non-proficient Bilinguals

**DOI:** 10.3389/fpsyg.2020.01414

**Published:** 2020-06-18

**Authors:** Xie Ma, Xiao Ma, Peng Li, Yan Liu

**Affiliations:** ^1^Faculty of Education, Yunnan Normal University, Kunming, China; ^2^Key Laboratory of Educational Information for Nationalities, Yunnan Normal University, Kunming, China; ^3^Faculty of Foreign Languages and Cultures, Kunming University of Science and Technology, Kunming, China

**Keywords:** emotional working memory, proficient bilinguals, non-proficient bilinguals, positive emotion, negative emotion

## Abstract

The impact of bilingual education and bilingual experience on working memory has been an important and controversial issue in the field of psycholinguistics. Taking Chinese-English bilinguals as an example, this study aims to investigate the differences in emotional working memory between proficient and non-proficient bilinguals by using delayed matching-to-sample task paradigm and the more complex *N*-back task in emotional contexts. The results show that proficient bilinguals may have better performance on both of these two working memory tasks than non-proficient bilinguals, and the advantage effects can be more apparent under high memory load conditions. In addition, the negative emotion context could have a positive impact on complex *N*-back tasks. This study supports the notion that bilingual experience can promote the development of an individual’s cognitive ability and enable individuals to possess more advantages in working memory even in the presence of emotional contexts.

## Introduction

Executive control is an essential and core cognitive function in goal-oriented behavior control and self-control, which are highly associated with cognitive development and sociality development (Hughes and Ensor, 2007; Best et al., 2009). In general, the executive control includes sub-functional components such as working memory, inhibition control, and cognitive switching (Jurado and Rosselli, 2007; Seeley et al., 2007).

Bilinguals are known as individuals who use and manage two languages, with the need to spontaneously select one of the languages according to the context and suppress the interference from non-goal language or non-attended language (Grosjean, 1992; Bialystok, 2017). The impact of bilingual education on executive control has been an important and controversial issue in the field of psycholinguistics (Morales et al., 2013; Ratiu and Azuma, 2015; Grundy and Timmer, 2017; Antoniou, 2019). A large number of studies have found that abundant bilingual experience is conducive to promoting the development of an individual’s abilities related to executive controls such as conflict resolution, cognitive switching, and memory storage; and this effect is called “Bilingual advantage” (Bialystok et al., 2010; Prior and MacWhinney, 2010; Morales et al., 2013; Bialystok, 2017; Scaltritti et al., 2017). For example, Bialystok et al. (2010) tested children aged 3 and 4 with word-mapping tasks and found that bilingual children showed stronger inhibitory control ability, which would become more significant as the children got more secondary language experience. Hernandez et al. (2013) conducted an experiment based on the intermittent clue transformation task, finding that when implicit clues were present, the restart cost of bilingual learners was lower than that of monolinguals, and that the bilinguals showed stronger switching ability.

By contrast, there were also a number of studies that did not confirm the cognitive advantage effects of bilingualism, among which the most controversial focus was the promoting effect of bilingual experience in working memory. Some studies have found that individuals with different bilingual experience showed no differences in performing working memory tasks (Bialystok et al., 2008; Bonifacci et al., 2011; Ratiu and Azuma, 2015). Moreover, individuals with more bilingual experience were in an inferior position for cognition development (Luo et al., 2013; Ratiu and Azuma, 2015). From samples of college students of Jing-po (one of the ethnic minority groups in China) and Lan et al. (2011) explored differences in performances of multiple executive control function tasks between those proficient in two or more dialects and those who only mastered one dialect. The results showed that the bilinguals had an advantage in both the tasks of inhibitory control (the stroop color-word task) and cognitive switching (the digit switching task), while the performances of two groups were not significantly different in working memory tasks (e.g., the memory and the updating task). In one study on working memory of bilinguals with different proficiency Kudo and Lee (2014) found that bilinguals proficient in a second language did not show any advantage in performing working memory tasks, such as phonological span and letter span, than those who were non-proficient. The meta-analysis based on previous studies demonstrated that the correlation between bilingual experience and working memory may be influenced by task patterns (Linck et al., 2014; Sullivan et al., 2016; Grundy and Timmer, 2017). In previous studies, some used simple working memory tasks, such as the span task, to investigate memory maintenance ability, while some others involved complex working memory tasks, such as the *N*-back task, to examine memory maintenance and updating ability. When task demands were relatively high, the promoting effect of bilingual experience on working memory tended to be significantly apparent (Linck et al., 2014; Sullivan et al., 2016). However, there is little research comparing the effects of bilingual experience on working memory by using different tasks. More studies are needed to demonstrate whether more bilingual experience can promote the development of working memory and whether the promoting effects are task-specific.

Emotional working memory is defined as the ability to successfully deploy working memory in emotional contexts, and is measured by the standard working memory task with an inserted emotional background (Schweizer et al., 2013; Barker and Bialystok, 2019). When compared with the standard or traditional task with no emotional background, emotional working memory task calls for higher requirements on cognitive operation (Schweizer et al., 2013; Janus and Bialystok, 2018; Bai et al., 2019; Katie et al., 2019) and can help examine whether individuals can resist the interference of emotion and effectively operate information processing in an emotional and social environment (Janus and Bialystok, 2018). Moreover, it can be used to assess individuals on their emotion regulation abilities, and at the same time, to predict their problem-solving abilities and social adaptation competence in the real-life situations (Ladouceur et al., 2009; Schweizer et al., 2013; Engen and Anderson, 2018; Janus and Bialystok, 2018).

In recent years, researchers have turned their attention to whether the facilitation effect of bilingual experience on standard or basic working memory can be transferred into emotional working memory (Janus and Bialystok, 2018; Barker and Bialystok, 2019). Bialystok et al. (2008) investigated the working memory ability of bilingual children aged 8 to 11 whose native languages were Portuguese, Filipino, Spanish, etc. with English being their second language, and compared their performances with English monolingual children. In their experiment, emotional *N*-back tasks were used, in which there were three emotional face (EF) contexts – anger, happiness, and neutrality. The results showed that the accuracy rate of bilingual children was significantly higher than that of monolingual children. Under the 1-back condition, no difference on the response time (RT) was found in the two groups. Under the 2-back condition, the RT of bilingual children was even higher than that of monolingual children. Based on the same experimental paradigm, Barker and Bialystok (2019) extended the research of Janus and Bialystok (2018) to study adult subjects, and they came to a similar conclusion. In these two studies, there were some contradictions between the implications of accuracy rate and RT, which made it difficult to accurately demonstrate whether the cognitive advantage effect of bilingual experience existed. In addition, these two studies only used the *N*-back task in emotional context, so it was difficult to decide whether the influence of bilingual experience on emotional working memory task was due to task-specificity. Furthermore, both studies made comparisons between participants with second language experience (bilingual) and participants with no experience (monolingual). It was highly likely that some other variables were involved, such as geography, economic status, and education background, etc. (Linck et al., 2014; Antoniou, 2019). The effect of bilingual experience can be observed more accurately if bilingual experience is used as a continuous variable (such as measuring the level of proficiency), that is, people with different levels of experience are supposed to be chosen (Linck et al., 2014; Novitskiy et al., 2019).

Extending on previous studies, this study further discusses the influence of bilingual experience on individuals’ emotional working memory by adopting the delayed matching-to-sample task paradigm (Experiment 1) and *N*-back task (Experiment 2) in the contexts of emotions. The delayed matching-to-sample task paradigm was the classical paradigm to investigate the ability to maintain working memory information (D’Esposito et al., 1999; Aben et al., 2012), while the *N*-back task paradigm was a more complicated task used to assess updating ability, in addition to the maintaining of information (Barker and Bialystok, 2019). In the process of performing these two tasks and experimental paradigms, it is possible for researchers to manipulate cognitive load to control the degree of difficulty of tasks. Referring to previous studies and experimental paradigms (Janus and Bialystok, 2018; Barker and Bialystok, 2019) this study added three kinds of conditions of face emotional contexts conditions – neutral emotion, negative emotion (sadness) and positive emotion (happiness) into the traditional working memory tasks, so as to explore the differences in working memory between bilingual people with different levels of proficiency based on the previous studies (Linck et al., 2014) under the three kinds of face emotional backgrounds.

The setting of neutral emotion condition in the present study could be used to demonstrate whether rich bilingual experience was conducive to promoting “basic” or “pure” working memory in individuals, and could be compared with the previous studies examining the influence of bilingual experience on standard or traditional working memory tasks (without emotional intervention). And the setting of emotional conditions (positive emotion and negative emotion) in the study would help bring about further discussions: whether the effect of bilingual experience on traditional working memory would be transferred to the influence on emotional working memory, and whether individuals with more bilingual experience could effectively resist the interference of EF stimulation and still maintain more advantages in working memory performance.

## Experiment 1: Emotional Delay-Matching-To-Sample Task

### Methods

#### Participants

All the participants who spoke Chinese as their native language and English as their second language were randomly selected from Yunnan Normal University. The data on their gender, growth environment and other information are shown in the Table 1. Twenty-six proficient bilinguals (*M* age = 23.0, *SD* = 1.3) majoring in English had passed TEM-8 (Test for English Majors-Band 8, the highest professional English test in China), and 31 non-proficient bilinguals (*M* age = 21.1, *SD* = 1.8) were non-English majors and they had not passed CET-4 (College English Test B and 4, the passing of which equals to a score of 5 in International English Language Testing System (China National Educational Examinations Authority, 2019) yet. Before the experiment, a set of assessment tests were conducted. All participants were asked to finish the Raven Test of Chinese version (Zhang and Wang, 1989) besides, participants were required to finish the SAS test (Self-Rating Anxiety Scale, Chinese version) (Wu, 1999) and SDS tests (Self-Rating Depression Sale, Chinese version) (Wang and Chi, 1984). Some researchers indicated that the depression or anxiety of the participants could have a certain impact on the processing of emotional stimulus (Nolen-Hoeksema, 2000; Joormann and Gotlib, 2008). As suggested, participants with a depression index score above 0.5 or an anxiety standard score above 50 did not meet the requirements of the present experiment. The score of each participant was under the standard score of SAS of 40, and the score of SDS was under 0.5. All participants volunteered to participate in the experiment and signed the agreement of informed consent. This study was approved by the local ethics committee of Yunnan Normal University, and it conformed to the ethical principles of the Declaration of Helsinki (World Medical Organization, 1996).

**TABLE 1:** The number of participants on gender, growth environment and English proficiency in experiment 1.

English proficiency	Gender			Growth environment		
	Female	Male	Total	Urban	Rural	Total
Proficient group	14	12	26	13	13	26
Non-proficient group	15	16	31	15	16	31
Total	29	28	57	28	29	57

#### Stimuli

The EF pictures were chosen from CAFPS (Chinese Affective Face Picture System) (Bai et al., 2005). All pictures were 260 × 300 pixels, normalized for size and luminance, including 30 negative (10 angry/10 sad/10 fear) EF pictures, 30 neutral (no emotion) EF pictures and 30 positive (happy) EF pictures. Numbers with two digits were randomly chosen from 10 to 99, three digits from 100 to 999, and four digits from 1,000 to 9,999.

#### Procedure

**FIGURE 1:**
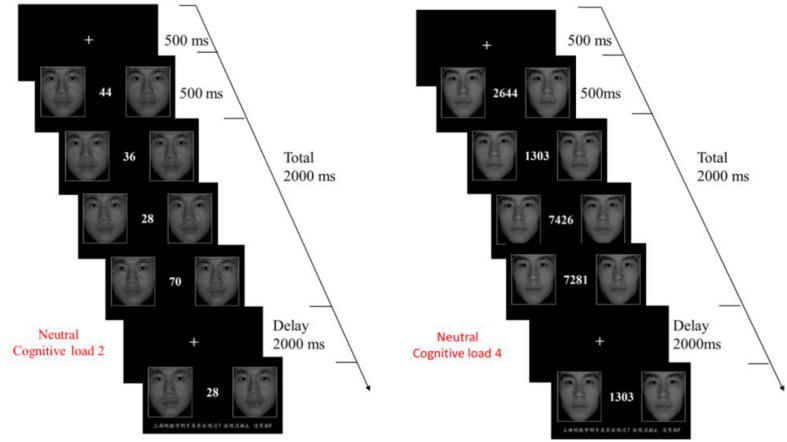
Emotional delay-match-to-sample task design. There were four groups of numbers displayed one after another, all of them cost 2,000 ms to finish. After that, a white fixation cross appeared again lasts for 2,000 ms, and then there were pictures with “numbers” in the center and EFs on both sides. The participants needed to judge whether the number had appeared in the previous four sets of numbers.

Figure 1 presents the procedure of the E-DMTS (emotional delay-matching-to-sample) task. The task was uniformly presented on a 19-inch LCD monitor with a resolution of 1,024 × 768 and a scanning frequency of 60 Hz, and was designed by Eprime 2.0. When the experiment began, a white fixation cross was displayed in the center of the black background screen for 500 ms, and then there were random numbers displayed in the center of the screen with same EF pictures on the both sides for 500 ms each time. The numbers ranged from 2 to 4 digits. There were four groups of numbers displayed one after another; all of them taking 2,000 ms. After that, a white fixation cross appeared again lasting for 2,000 ms, and then there were pictures with “numbers” in the center and EF on both sides. The participants needed to judge whether the numbers had appeared in the previous four sets of numbers. If the numbers were the same as the previous four sets of numbers, participants needed to press the “F” key. Otherwise, they pressed the “J” key. In practice, only when the accuracy was more than 80% could the participants got into the formal experiment. There were a total of three blocks, each represented an emotional type, consisting of 30 trials (i.e., 15 target trials and 15 non-target trials). The 30 trials contained three types of memory loads, each with 10 trials.

### Results

*T*-test for language group showed no differences between the proficient group and non-proficient group on Raven test scores (*M* proficient group = 51.62, *SD* proficient group = 3.25, *M* non-proficient group = 50.65, *SD* non-proficient group = 3.5, *t* = 1.077, and *p* = 0.286). The result also showed that the differences between proficient group and non-proficient group on SAS test were not significant (*M* proficient group = 29.27, *SD* proficient group = 3.13, *M* non-proficient group = 28.65, *SD* non-proficient group = 2.47, *t* = 0.841, and *p* = 0.404), and the differences between the two groups on SDS test were not significant (*M* proficient group = 0.351, *SD* proficient group = 0.037, *M* non-proficient group = 0.361, *SD* non-proficient group = 0.043, *t* = −0.955, and *p* = 0.441).

The data of accuracy in different conditions are shown in Table 2. Accuracy was analyzed by using a three-way ANOVA for bilingual experience groups (proficient and non-proficient), emotional conditions (positive, neutral, and negative), and memory load conditions (two, three, and four digits). For all analyses, the degrees of freedom of the *F* ratio were corrected for violations of the sphericity assumption based on the Greenhouse Geisser correction (Greenhouse and Geisser, 1959) and Bonferroni corrections were used for each comparison (Keppel, 1991). The results showed a main effect of language groups [*F* (1, 55) = 17.79, *p* < 0.001, and $\eta_{p}^{2}$ = 0.24], the accuracy of proficient bilinguals (86.6%) was higher than that of non-proficient bilinguals (78.7%). A main effect of memory load conditions was also found [*F* (2, 54) = 50.79, *p* < 0.001, and $\eta_{p}^{2}$ = 0.48], with memory loads increasing and accuracy decreasing, in the order of two digits (89.6%) > three digits (80.7%) > four digits (77.8%). The interaction between language groups and memory load conditions was significant [*F* (2, 54) = 3.64, *p* < 0.05, and $\eta_{p}^{2}$ = 0.06]. Further analysis revealed that the differences in language groups was significant under three digits condition (*p* = 0.006), and under four digits condition (*p* < 0.001).

**TABLE 2:** Mean score and standard deviation for accuracy on E-DMTS task by different language groups [M (SD)%].

Language type	Emotion type	Memory load		
		Two digits	Three digits	Four digits
Proficient	Positive	91.92 (10.21)	85.00 (13.03)	83.85 (14.71)
	Neutral	93.46 (7.97)	82.31 (10.70)	85.77 (11.72)
	Negative	91.15 (10.70)	85.00 (12.73)	81.15 (12.43)
Non-proficient	Positive	84.84 (13.87)	77.74 (14.07)	70.32 (16.01)
	Neutral	88.06 (10.13)	78.40 (15.51)	73.55 (14.27)
	Negative	88.06 (10.78)	75.48 (13.38)	72.26 (17.27)

**FIGURE 2:**
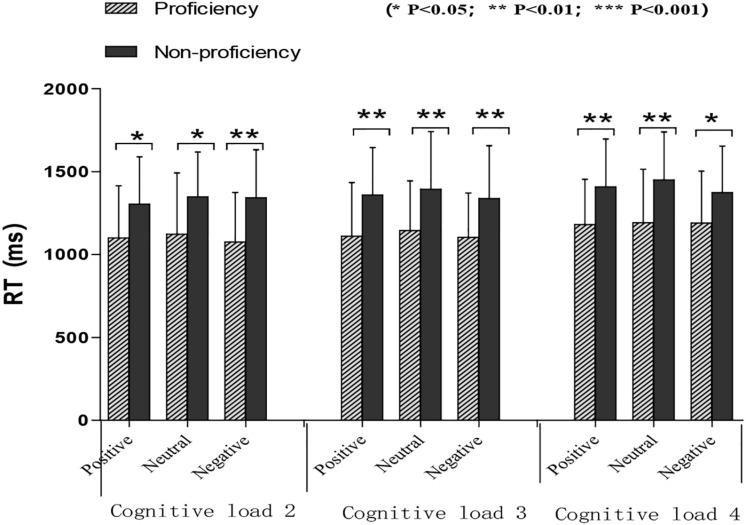
Mean score and standard deviation for reaction time on emotional delay-matching-to-sample task by different language groups by different language groups. The reaction time of proficient bilinguals was shorter than that of non-proficient bilinguals, and the difference was significant in all conditions.

The data of reaction time in different conditions are shown in Figure 2. RT was also analyzed by using a three-way ANOVA for bilingual experience groups, emotional conditions, and memory load conditions. The results revealed a main effect of language groups [*F* (1, 55) = 11.47, *p* < 0.01, and $\eta_{p}^{2}$ = 0.17], with RT of the proficient (*M* = 1,135.57 ms, *SD* = 50.70) shorter than that of the non-proficient (*M* = 1,368.34 ms, *SD* = 46.43). Also a main effect was found in memory load conditions [*F* (2, 54) = 13.01, *p* < 0.001, and $\eta_{p}^{2}$ = 0.19], that is, two digits (*M* = 1,215.47 ms, *SD* = 35.77) <3 digits (*M* = 1,241.56 ms, *SD* = 35.45) <4 digits (*M* = 1,298.84 ms, *SD* = 35.89). A correlation was computed between accuracy and RT to determine whether there were speed-accuracy trade-offs. The relation was significant, but was negative [*r*(57) = −0.34, *p* < 0.05].

### Discussion

Results of delayed matching task in the present study uncovered that compared with non-proficient bilinguals, proficient bilinguals performed better with higher accuracy and shorter RT in the non-emotional context (i.e., under neutral emotion condition). That is, the proficient bilinguals showed significant advantages in maintaining representations of information (i.e., digital information retention was the focus in the present study), which could be supported by some previous studies on the relationship between bilingual experience and information retention (e.g., the memory span task) (Tzou et al., 2012; Morales et al., 2013). For examples, Morales et al. (2013) reported the performance of monolingual and bilingual children on visuo-spatial span task through simultaneous or sequential presentation of stimulus. The task was also required for maintaining information, and the result demonstrated that bilinguals outperformed monolinguals overall. Additionally, the present study has extended these results and found that under emotional background conditions (i.e., pictures with positive emotions and negative faces), proficient bilinguals also had better performances, that proficient bilinguals showed shorter RTs- than non-proficient bilinguals with both positive and negative face emotions. The present study indicated that it was possible that the promotion effect of bilingual experience on information retention under non-emotional conditions could be transferred to promote the retention of information in the emotional contexts.

However, the results showed a significant interaction between bilingual experience and memory load. The advantage effect of bilingual experience reached a statistically significant level only under high memory load conditions (memory load of three and four digits), while it was not significant under low memory load conditions (memory load of two digits). These results indicated that the advantage effect in proficient bilinguals could be more obvious, under the circumstance of large amount of information to be maintained. The previous meta-analysis also suggested that the cognitive advantage brought by bilingual experience was influenced by the difficulty of task (Bialystok and Feng, 2009; Bonifacci et al., 2011; Sullivan et al., 2016) and the performances of bilinguals on simple and low-load working memory tasks, in most cases, showed no more significance than monolinguals (Yang et al., 2005). Researchers speculated that the low-level difficulty of tasks involving less attention resources could lead to “ceiling effect,” which would conceal the advantage of bilinguals (Costa et al., 2008; Morrison et al., 2018; Prior and MacWhinney, 2010). For example, some studies investigating the effect of bilingual experience on inhibitory ability suggested that the advantage of bilingual experience seemed to be more obvious in the Stroop task than that in the Flanker task. Researchers explained that the main reason was probably due to the fact that the Stroop task was more difficult and involved more attention resources (MacLeod and MacDonald, 2000; Bialystok et al., 2014).

## Experiment 2: Emotional *N*-Back Task

### Methods

#### Participants

The participants are the same as in Experiment 1. However, because the accuracy on for three non-proficient bilinguals were so low that they did not passed the practice phrase and got into the formal experiment, their data were dropped out. The final data came from 26 proficient bilinguals (*M* age = 23.0, *SD* = 1.3) and 28 non-proficient bilinguals (*M* age = 21.4, *SD* = 1.6). The data on their gender, growth environment and other information are shown in the Table 3.

**TABLE 3:** The number of participants on gender, growth environment, and English proficiency in experiment 2.

English proficiency	Gender			Growth environment		
	Female	Male	Total	Urban	Rural	Total
Proficient	14	12	26	13	13	26
Non-proficient	14	14	28	14	14	28
Total	28	26	54	27	27	54

#### Material

Nine EF pictures were added, including each emotional type for three based on pictures of Experiment 1. Since some letters were easily confused, the letters with similar shapes were removed. The letters were chosen as follows: A, B, E, G, H, K, L, M, N, Q, R, S, W, X, and Z.

#### Procedure

**FIGURE 3:**
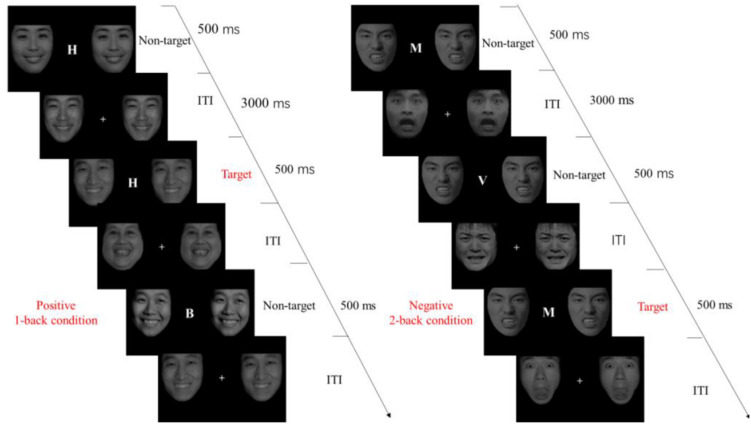
Emotional *N*-back task design. When the experiment began, a white fixation cross was displayed in the center of the screen for 500 ms, and then a random letter was displayed in the center of the screen with same EF pictures on both sides for 500 ms. After 3,000 ms another picture with a letter in the center and EF pictures on both sides were displayed for 500 ms, and participants were asked to judge whether this letter was the same with the letter they saw one screen back (1-back) or two screens back (2-back).

The procedure of the E-*N*-back (Emotional *N*-back) task was presented in Figure 3. When the experiment began, a white fixation cross was displayed in the center of the screen for 500 ms, and then a random letter was displayed in the center of the screen with same EF pictures on both sides for 500 ms. After 3,000 ms another picture with a letter in the center and EF pictures on both sides were displayed for 500 ms, and participants were asked to judge whether this letter was the same with the letter they saw one screen back (1-back) or two screens back (2-back). If it was the same, they were asked to press F on the keyboard, otherwise they were asked to press J. They were asked to respond quickly and accurately. Unless the accuracy of participants was more than 80% in practice, they couldn’t get into the formal experiment. There was also a group of practices prior to the beginning of 2-back condition. There were three three blocks in 1-back condition including three emotional types, where each block consisted of 15 target trials and 16 non-target trials. Except non-target trials that were increased to 17 trials, others under 2-back condition were the same as under 1-back condition. It would cost about 20 min to finish the total task.

### Results

Since the data from three non-proficient were dropped out. The differences between language group were analyzed again using *T*-test. The results showed there were no significant differences between the proficient group and non-proficient group on Raven Test scores (*M* proficient group = 51.62, *SD* proficient group = 3.25, *M* non-proficient group = 50.43, *SD* non-proficient group = 3.52, *t* = 1.284, *p* = 0.205). The result also showed that the differences between proficient group and non-proficient group on SAS test were not significant (*M* proficient group = 29.27, *SD* proficient group = 3.13, *M* non-proficient group = 28.68, *SD* non-proficient group = 2.57, *t* = 0.76, *p* = 0.451), and the differences between the two groups on SDS test were not significant (*M* proficient group = 0.351, *SD* proficient group = 0.037, *M* non-proficient group = 0.364, *SD* non-proficient group = 0.042, *t* = −1.229, *p* = 0.225).

The data for accuracy in different conditions are shown in Table 2. The data were analyzed using a three-way ANOVA for bilingual experience groups (proficient/non-proficient) as a between participant variable, treating emotional conditions (positive/neutral/negative) and *N*-back conditions (1-back/2-back). The results indicated a main effect of *N*-back conditions [*F* (1, 52) = 42.86, *p* < 0.001, and $\eta_{p}^{2}$ = 0.45], and the accuracy under 1-back condition (94.8%) was higher than that under 2-back condition (88.1%). A main effect of emotional conditions was found [*F* (2, 51) = 6.48, *p* < 0.01, and $\eta_{p}^{2}$ = 0.11], with the accuracy of positive emotion (90.0%) slightly lower than of neutral emotion (91.9%) and negative emotion (92.4%). The interaction between emotional conditions and *N*-back conditions was significant [*F* (2, 51) = 4.06, *p* < 0.05, and $\eta_{p}^{2}$ = 0.072]. The further analysis showed that, under 1-back condition, the accuracy of positive emotion was lower than that of neutral emotion (*p* < 0.01), under 2-back condition, the accuracy of positive emotion was lower than of negative emotion (*p* < 0.01).

**FIGURE 4:**
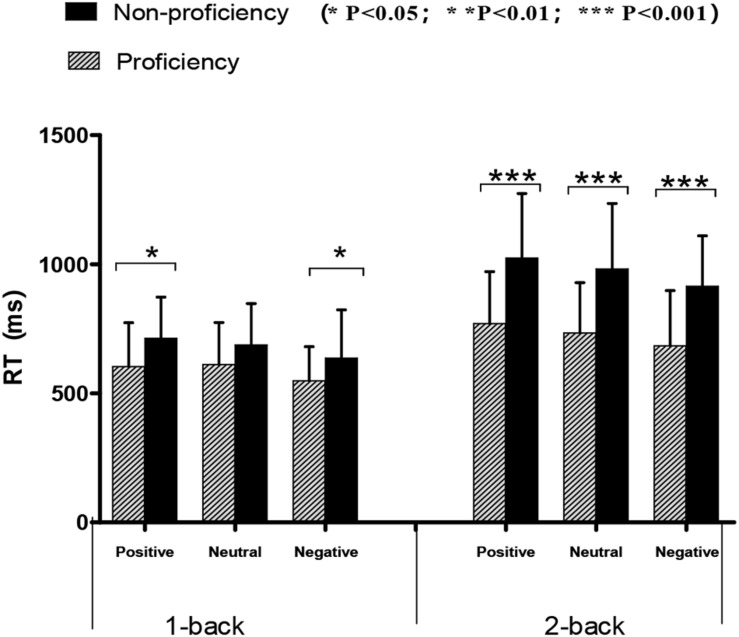
Mean score and standard deviation for RTs on emotional *N*-back task by different language groups. The RTs of proficient bilinguals was shorter than that of non-proficient bilinguals, and the difference was significant in most conditions beside in negative/1-back (marginal significant, *p* = 0.056) and in neutral/1-back conditions.

The data of RT under different conditions are shown in Figure 4, and were analyzed using a three-way ANOVA. A main effect was found in language groups [*F* (1, 52) = 15.42, *p* < 0.001, and $\eta_{p}^{2}$ = 0.23], and proficient bilinguals (*M* = 658.577 ms, *SD* = 30.71) performed better than non-proficient bilinguals (*M* = 826.03 ms, *SD* = 29.60). The results also indicated a main effect of *N*-back conditions [*F* (1, 52) = 98.98, *p* < 0.001, and $\eta_{p}^{2}$ = 0.66], and participants performed better under 1-back condition (*M* = 633.26 ms, *SD* = 20.60) than under 2-back condition (*M* = 851.35 ms, *SD* = 26.93), and a main effect of emotional condition was found [*F* (2, 51) = 17.63, *p* < 0.001, and $\eta_{p}^{2}$ = 0.25], the RT in negative emotion context (*M* = 695.97 ms, *SD* = 22.06) was shorter than in neutral (*M* = 753.3 ms, *SD* = 23.19) and positive emotion contexts (*M* = 777.64 ms, *SD* = 23.22). The interaction between language groups and *N*-back conditions was significant [*F* (1, 52) = 12.23, *p* < 0.01, and $\eta_{p}^{2}$ = 0.19]. Further analysis indicated that the difference between proficient and non-proficient groups under 2-back condition was more significant than the difference under 1-back condition. A correlation was computed between accuracy and RT to determine whether there were speed-accuracy trade-offs. There were no significant correlations [*r*(54) = −0.007, *p* > 0.05].

### Discussion

The *N*-back task calls for more complex requirements on working memory than the delayed matching task, particularly since it involves examining the functions of updating information as well as of storing and retaining information (Liu et al., 2017; Janus and Bialystok, 2018; Barker and Bialystok, 2019). Experiment 2 adopted emotional *N*-back tasks and simultaneously manipulated the emotional backgrounds (positive/neutral/negative) and memory load (1-back/2-back). Such results revealed that the proficient bilinguals showed shorter RT than non-proficient bilinguals without emotions (neutral condition), indicating an advantageous effect in proficient bilinguals. What’s more, proficient bilinguals also showed advantageous effects in the emotion conditions (positive/negative). That is to say, proficient bilinguals had shorter RT than non-proficient bilinguals when they carried out the *N*-back task under both positive and negative emotion conditions. Using similar emotional *N*-back tasks, recent studies examined differences between bilingual children whose mother tongues are Portuguese, Philippine or Spanish with English as second language and monolingual children who speak English. The results showed that, in terms of accuracy, bilingual children were significantly more accurate than monolingual children. Regarding reaction time, however, bilingual children presented significantly slower responses than bilingual children under the 2-back condition, and there were speed-accuracy trade-offs (Janus and Bialystok, 2018). Compare with the previous study by Janus and Bialystok (2018). The prompting effect of the bilingual experience in present study might be more obvious and certain, because compare to the non-proficient bilinguals, the proficient bilinguals had a shorter RT with the similar accuracy, meanwhile, the trade-offs of speed-accuracy were insignificant. It was speculated that the disparity in participants’ age was considered to account for this finding. Generally, the adult population is likely to have higher and more stable working memory than children, and may perform better with less RT (Stoltzfus et al., 1996) simultaneously may not have to give away the accuracy. Besides, the gaps between the native and non-native languages were thought to be another reason. Bilinguals in the present study spoke Chinese and English. As is known, there are huge differences between these two languages in such aspects as forms, phonetic rules, process of semantic extraction, etc. (Pan, 1997). Such great differences might yield higher demands for working memory in the process of non-native language learning and more training of working memory skills (Yu et al., 1985; Lan et al., 2011). Accordingly, it was possible that with many years of learning, the capacity of working memory would be improved significantly.

Moreover, the results of Experiment 2 suggested that the cognitive advantage of more bilingual experience was affected by memory load. Although proficient bilinguals either in the 1-back task or the 2-back task showed shorter reaction time than non-proficient bilinguals, it should be noted that the difference of reaction time between proficient bilinguals and non-proficient bilinguals in the 2-back task was significantly greater than that in the 1-back task. That is, with the task becoming more difficult, the gap between proficient bilinguals and non-proficient bilinguals was seemingly widened. It was speculated that non-proficient bilinguals were at a disadvantage in information processing, and they, with a high memory load, might be more susceptible to disturbance by some distractions and interference factors (Bialystok and Feng, 2009; Prior and MacWhinney, 2010; Chen and Wang, 2018). Thus, there could emerge a larger lagging effect in non-proficient bilinguals.

In addition, this study found that the background information with negative emotions could improve individuals’ performance in such *N*-back working memory tasks, especially under the condition of high memory load. To be more specific, under the 2-back condition, the promoting effect of negative emotions was significantly greater than that of positive emotions and neutral emotions, and this result could consist with accumulating evidence based on previous studies (Luo et al., 2014; Zhang et al., 2017). A typical example of this was Grimm et al. (2012) who took advantage of the emotional *N*-back task to explore the differences in the functions of updating under different emotional expressions, revealing that negative emotion words significantly facilitated the participants for their reaction on working memory under the 2-back condition.

## General Discussion

There have been controversial discussions on whether the promotion effect of bilingual experience in executive control function could be reflected in sub-component working memory (Chen and Wang, 2018; Antoniou, 2019). Some studies did not support that individuals with different bilingual experience would have different performances in the tasks involving working memory (Bialystok et al., 2008; Bonifacci et al., 2011; Ratiu and Azuma, 2015; Wang et al., 2017). However, the present study found that bilingual education or bilingual training has an important promoting effect on working memory; the proficient bilinguals seemed to have better performance than non-proficient bilinguals, whether in delayed matching task or in the more challenging *N*-back tasks. Researchers formulated two reasons to explain why working memory could be enhanced by bilingual learning. Firstly, from the perspective of unity, the main functions of bilingual training were to achieve free switching between different languages and to avoid the interference of non-target language (Scaltritti et al., 2017; Tao et al., 2017) but the established effect of bilingual training on switching or inhibition components of the executive control system will necessarily involve working memory through some common cognition foundation among components of the system. Secondly, from the perspective of diversity, the joint activation of both languages in language processing requires not only inhibition and transformation but also retention of more representations of context, interlocutors, and discourse referring to two languages – all functions of working memory. As a result, rich bilingual experience was likely to present benefits for the working memory (Bialystok et al., 2012; Morales et al., 2013; Costa and Sebastián-Gallés, 2014).

Besides, the present study showed that the individuals with rich bilingual experience still had better performance in more complicated conditions involving emotional background, with both the traditional delayed matching task and *N*-back tasks. Considering that the capacity of emotional working memory could embody emotion regulation in the interaction process of emotions and cognition (Ladouceur et al., 2009; Engen and Anderson, 2018; Janus and Bialystok, 2018) and to further predict social adaptation (Bryson et al., 1999; Bowie and Harvey, 2007; Schweizer et al., 2013) the results of the current study potentially indicated that bilingual training could have a more profound and comprehensive impact on individual development. It may involve not only the shaping of “pure” or “basic” cognitive functions, but also the shaping of some “social” cognitive functions, including emotion regulation, social adaptation and so on. However, future researches should directly explore the relationship between bilingual experience and some capabilities of social cognition, and provide more direct and firm evidences for the relationships.

It is noteworthy that the two experiments generated some distinct findings. The main effect of emotion only appeared in Experiment 2. Compared with the delayed sample matching task, the *N*-back task not only investigated the retention and storage of working memory, but also examined the updating function of information. The results suggested that the influence of emotional condition on working memory might also be affected by cognitive complexity. That is, the more complex the tasks were, the greater the influence it could have. Complex tasks were inclined to involve higher cognitive load, and the association between emotion and working memory could be adjusted by cognitive load based on a previous study. Erk et al. (2007) used Sternberg item recognition paradigm to study the influence of emotional background in working memory under different cognitive loads. The results showed that under the condition of high-load working memory, negative emotions improved the performance of working memory, whereas under the condition of low-load working memory, the influence of emotional stimuli and neutral stimuli on working memory had no significant difference (Erk et al., 2007). This suggested that the effect of emotion on working memory was not stable, and it might change with task complexity and cognitive load.

## Conclusion

Involving Chinese-English bilinguals as participants, this study examined the differences in emotional working memory between proficient and non-proficient bilinguals by adopting delayed matching-to-sample task paradigm and the more complex *N*-back task in emotional contexts. The results showed that proficient bilinguals have better performance on both of the two working memory tasks than non-proficient bilinguals, and the advantageous effects were more obvious under the conditions of high memory load. Besides, negative emotion has an activated impact on complex *N*-back tasks. The results suggests that abundant bilingual experience can promote individual development of cognitive ability and enable individuals to possess cognitive advantages on working memory, and even in the presence of emotional contexts. The present results advance the understanding of how bilingualism impacts working memory and greatly support the view of “bilingual cognitive advantage” (Bialystok, 2017; Scaltritti et al., 2017). However, the potential influences of bilingual education are more profound and far-reaching, and the benefits of bilingual experience on capacities of social cognition involved emotional working memory should be more investigated and discussed in the future study.

## Data Availability Statement

The datasets generated for this study are available on request to the corresponding author.

## Ethics Statement

The studies involving human participants were reviewed and approved by the local Ethics Committee of Yunnan Normal University. The patients/participants provided their written informed consent to participate in this study. Written informed consent was obtained from the individual(s) for the publication of any potentially identifiable images or data included in this manuscript.

## Author Contributions

XieM and XiaoM proposed the experiment, designed the procedure, and did the most work of the manuscript. YL gave useful comments to the experiment and helped to revise the manuscript for several times. PL helped to find participants and did the experiment work. All authors contributed to the article and approved the submitted version.

## Conflict of Interest

The authors declare that the research was conducted in the absence of any commercial or financial relationships that could be construed as a potential conflict of interest.
